# Effect of Combining Conventional and Telehealth Methods on Managing Peritoneal Dialysis Patients: A Retrospective Single-Center Study

**DOI:** 10.1155/2022/6524717

**Published:** 2022-03-03

**Authors:** Zhi Wang, Wenjing Yan, Ying Lu, Kai Song, Huaying Shen, Yun Wang, Sheng Feng

**Affiliations:** ^1^Department of Nephrology, Second Affiliated Hospital of Soochow University, Suzhou, China; ^2^Department of Nursing, Second Affiliated Hospital of Soochow University, Suzhou, China

## Abstract

**Objective:**

This study aimed to explore follow-up mode changes for peritoneal dialysis (PD) patients and their effects on PD quality during the COVID-19 pandemic.

**Methods:**

A retrospective single-center study was conducted. All patients who received PD treatment at the Second Affiliated Hospital of Soochow University between January 2018 and March 2020 were enrolled in this study. Patient data during the first quarter of 2018 (Q1-2018), the first quarter of 2019 (Q1-2019), and the first quarter of 2020 (Q1-2020) were collected.

**Results:**

No significant differences were observed for any serum examinations in different follow-up periods (*P* > 0.05). A significantly reduced outpatient follow-up rate was observed in Q1-2020 compared with Q1-2018 and Q1-2019 (71.6% Vs 78.9% Vs 84.7%, *P* < 0.001), accompanied by a significantly increased remote follow-up rate (28.4% Vs 21.1% Vs 15.3%, *P* < 0.001). Compared with Q1-2018 and Q1-2019, the hospitalization rate (27.7% Vs 30.9% Vs 15.7%, *P* < 0.001) and the incidence of peritonitis (0.162 Vs 0.186 Vs 0.08 per patient-year, *P* < 0.001) decreased significantly in Q1-2020. PD patients had a significant decline in the drop-out rate for Q1-2020 compared with Q1-2019 (4.4% Vs 7.3% Vs 2.2%, *P* < 0.001). No differences in the incidence of catheter-related infections were observed. No significant differences were observed for any peritoneal dialysis key performance indicators (KPIs) between outpatient follow-up and remote follow-up patients.

**Conclusion:**

During the COVID-19 pandemic (Q1-2020), our center practiced more remote follow-up procedures in PD patients. The hospitalization rate and peritonitis incidence were significantly decreased compared with the same time in previous years. No statistical differences were observed in other KPIs for peritoneal dialysis. This study shows that telehealth methods are a reasonable alternative to in-person care in the care/management of PD patients.

## 1. Introduction

In December 2019, several viral pneumonia cases with unknown etiology were reported in Wuhan, Hubei Province, China. This disease was named coronavirus disease 2019 (COVID-19) after deep-sequencing analyses of lower respiratory tract samples indicated the presence of a novel coronavirus [1]. COVID-19 spreads rapidly to all 34 provincial-level administrative regions in China, resulting in the decision to classify this disease as a Class B infectious disease, in compliance with the Law of the People's Republic of China on Prevention and Treatment of Infectious Diseases; however, the preventive and control measures for a Class A infectious disease were implemented. This decision was followed by the World Health Organization (WHO) declaration that this Chinese outbreak represented a Public Health Emergency of International Concern. The virus wreaked havoc throughout the country, reaching its first epidemic peak between 24 and 26 January 2020 [2]. Jiangsu was one of the 10 Chinese provinces most affected by COVID-19 from 22 January 2020 to 31 March 2020 [3]. Many large-scale public hospitals were forced to launch new epidemic policies promptly, including the Second Affiliated Hospital of Soochow University, where the Peritoneal Dialysis Center immediately implemented policy advocacy measures.

Peritoneal dialysis (PD) patients are susceptible to COVID-19, and the infection of COVID-19 worldwide in peritoneal dialysis patients has been mentioned in many available literature [4–6]. Amidst this national fight against COVID-19, material changes were made to the types of follow-up visits and the lifestyles of patients treated with peritoneal dialysis; at this time, however, no relevant literature has been reported regarding the impacts of COVID-19 measures on the quality of peritoneal dialysis. Therefore, this study was designed to provide a retrospective analysis of the general data, serological indicators, hospitalization rate, drop-out rate, infection-related complications, and other related prognostic factors for eligible patients treated at the Second Affiliated Hospital of Soochow University to investigate any changes in the key performance indicators (KPIs) for peritoneal dialysis during the COVID-19 epidemic.

## 2. Data and Methods

### 2.1. Subjects

All patients with complete follow-up data who received regular peritoneal dialysis (including newly catheterized patients) at the Peritoneal Dialysis Center of the Second Affiliated Hospital of Soochow University from January 2018 to March 2020 were enrolled in this study. Before the formal commencement of continuous ambulatory peritoneal dialysis, all patients received a Tenckhoff catheter using a twin-bag system. Additionally, all patients and their dialysis operators participated in standard training courses at the Peritoneal Dialysis Center and were assessed as qualified.

The Ethics Committee approved the study protocol of the Second Affiliated Hospital of Soochow University. Due to the study's retrospective nature, informed written consent was waived, and informed consent was not obtained.

### 2.2. Research Contents

The sociodemographic data for all patients were pooled, including gender, age, body mass index (body mass (kg)/height (m^2^)), and the duration of peritoneal dialysis. The peritoneal dialysis KPIs, including hemoglobin, total calcium, intact parathyroid hormone (iPTH), albumin, urea, creatinine, uric acid, and potassium levels, follow-up type, hospitalization rate, drop-out rate, the incidence of peritonitis, and the incidence of catheter-related infection, were collected for patients treated during the first quarter of 2018, the first quarter of 2019, and the first quarter of 2020.

Every PD patient has a record in our hospital. Remote follow for PD patients contains their weight, urine volume, ultrafiltration volume, diet, blood pressure, edema, fatigue, sleep, PD catheter exit conditions, change of PD prescription and drugs, etc. Discomfort and complication will be recorded if they happen. Follow-up frequency for remote PD patients was at least every 1–3 months.

### 2.3. Definition of Peritonitis, Recurrent Peritonitis, Catheter Bag Port, and Tunnel Infection

Peritonitis was defined by the presence of at least 2 of the following: (1) the clinical features of peritonitis, such as abdominal pain and/or cloudy dialysis effluent; (2) white blood cell (WBC) count in the dialysis effluent >100/*μ*L or  > 0.1 × 10^9^/L (dwell at least 2 hours), with polymorphonuclear leukocytes (PMN) > 50% of all leukocytes; and (3) positive effluent culture [7].

An infection with the same organism or a culture-negative episode within four weeks of a prior episode was defined as recurrent peritonitis.

An exit-site infection is usually indicated by the presence of purulent drainage, with or without skin erythema at the catheter-skin interface. In contrast, a tunnel infection may present as erythema, edema, or tenderness over the subcutaneous tract that may be clinically undetectable and often occurs concurrently with exit-site infections. These infections are often referred to collectively as catheter-related infections [7].

### 2.4. Definition and Calculation of Indicators

The outpatient follow-up rate was calculated by dividing the number of patients followed up during a given period by the total number of patients enrolled during the same period and is reported as *n* (%), representing the number of outpatient follow-up visits per 100 patient-years.

(1) $$\begin{array}{c} \text{Outpatient follow}‐\text{up rate}=\frac{\text{number of patients followed up}}{\text{the total number of patients enrolled}}*100\%. \end{array}$$

The remote follow-up rate was calculated by dividing the sum of telephone and Internet follow-up visits during a given period by the total number of patients during the same period and is expressed as *n* (%), representing the number of remote follow-up visits per 100 patient-years.

(2) $$\begin{array}{c} \text{Remote follow}‐\text{up rate}=\frac{\text{number of patients remote followed up}}{\text{the total number of patients enrolled}}*100\%. \end{array}$$

The hospitalization rate was calculated as the annual number of residents in a defined area hospitalized with a positive SARS-CoV-2 laboratory test divided by the number of patients classified as critical within that defined area [8], expressed as *n* (%) hospitalizations per 100 patient-years [9].

The drop-out rate was calculated as the annual number of people who discontinued peritoneal dialysis divided by the total number of peritoneal dialysis patients for the same year and is expressed as *n* (%), representing the number of drop-outs per 100 patient-years.

The incidence of peritoneal dialysis-related infections (peritonitis and catheter-related infections involving any microorganism) was calculated as follows: (1) the total number of patient-months on peritoneal dialysis is divided by the number of episodes of peritonitis and is expressed as the number of months between episodes; (2) the number of microbial infections in a given period is divided by the number of patient-years on peritoneal dialysis and is expressed as the number of annual episodes, with peritonitis relapse counted as an episode. This value is recorded as the number of episodes per patient per year (per patient-year) [10].

### 2.5. Treatment of PD Patients Who Had Emergencies

Patients who had PD-associated peritonitis were treated at the outpatient department according to ISPD guidelines. If COVID-19 nucleic acid test results were negative, the patients would receive in-hospital treatment. PD patients who had other emergencies were treated at emergency until the COVID-19 nucleic acid test results were negative.

### 2.6. Statistical Approach

The sociodemographic data and serological indicators were examined for normal distributions and homogeneity of variance. Continuous variables with normal distributions were presented as means ± SD, and differences among groups were tested by multivariate analysis of variance and *t*-test. ANOVA has been used in the comparison of serological indicators. If there was statistical significance among groups, then make a pairwise comparison afterward. Count data, including follow-up rate, hospitalization rate, and drop-out rate, were presented as percentages (%). Incidence of peritonitis and catheter-related infection rates were presented as per patient-year. Categorical data were compared with the chi-squared test. Fisher's precision probability test was used if less than five events were observed. All computations were performed using SPSS 25.0 for Windows (SPSS Inc., Chicago, IL, USA), and *P* < 0.05 was considered statistically significant.

## 3. Results

### 3.1. Comparison among Q1-2018, Q1-2019, and Q1-2020 Groups

#### 3.1.1. Comparison of General Condition Serum Examination among Q1-2018, Q1-2019, and Q1-2020 Groups

The clinical data of PD patients in Q1-2018, Q1-2019, and Q1-2020 groups are shown in Tables 1 and 2. Descriptive statistics were all in a normal distribution. No significant differences were observed for clinical data, such as gender, age, and body mass index among patients in the Q1-2018, Q1-2019, and Q1-2020 groups (*P* > 0.05). A significant variation was observed for the duration of peritoneal dialysis (*P* < 0.05), as demonstrated by the dramatically longer mean duration observed for the Q1-2020 group (52.0 ± 39.0 months) compared with durations of 43.8 ± 33.4 months in the Q1-2018 group and 50.3 ± 35.7 months in the Q1-2019 group (Table 1). No significant differences were observed for any blood indexes among patients in the Q1-2018, Q1-2019, and Q1-2020 groups (all *P* > 0.05, Table 2).

#### 3.1.2. Changes in Follow-Up Visit Types between Q1-2018, Q1-2019, and Q1-2020

**Figure 1:**
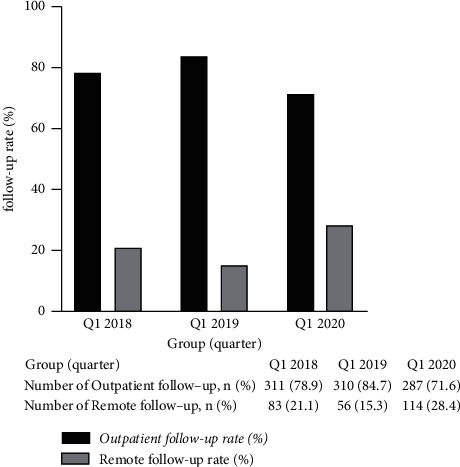
Changes in follow-up types (Q1-2018: the first quarter of 2018; Q1-2019: the first quarter of 2019; Q1-2020: the first quarter of 2020).

In Q1-2018 and Q1-2019, the reasons for remote follow-up were because patients live in different cities and barriers in transfer to our PD center. Remote follow-up was also used in patients with low compliance. The Q1-2020 period was associated with a significantly reduced outpatient follow-up rate, compared with Q1-2018 and Q1-2019 (*χ*^2^ = 5.780, *P* < 0.05; *χ*^2^ = 19.117, *P* < 0.001, respectively), which was accompanied by a significant increase in the remote follow-up rate (*χ*^2^ = 5.780, *P* < 0.05; *χ*^2^ = 19.117, *P* < 0.001, respectively, Figure 1).

#### 3.1.3. Comparison of Hospitalization Rates and Drop-Out Rates among Q1-2018, Q1-2019, and Q1-2020

17, 27, and 9 subjects ceased PD in Q1-2018, Q1-2019, and Q1-2020, respectively. In Q1-2018, nine subjects died, five subjects were transferred to hemodialysis, five subjects underwent renal transplantation, and one subject ceased PD because of renal function recovery. In Q1-2019, twelve subjects died, nine subjects were transferred to hemodialysis, five subjects underwent renal transplantation, and one subject ceased PD because of renal function recovery. In Q1-2020, seven subjects died, one subject was transferred to hemodialysis, and one subject underwent renal transplantation.

**Figure 2:**
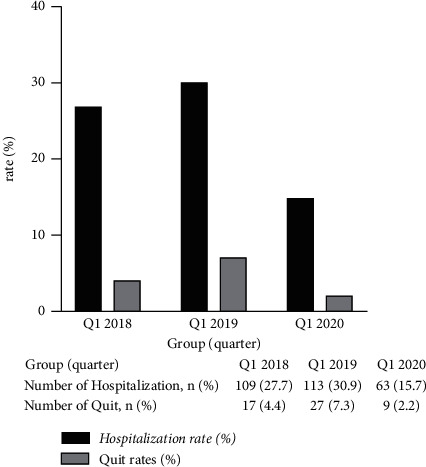
Comparison of hospitalization rates and drop-out rates (Q1-2018: the first quarter of 2018; Q1-2019: the first quarter of 2019; Q1-2020: the first quarter of 2020).

The Q1-2020 period was associated with a significantly reduced hospitalization rate, compared with Q1-2018 and Q1-2019 (*χ*^2^ = 16.751, *P* < 0.001; *χ*^2^ = 24.884, *P* < 0.001, respectively), and a significantly decreased drop-out rate compared with Q1-2019 (*χ*^2^ = 11.848, *P* < 0.05, Figure 2).

#### 3.1.4. Comparison of the Incidence of Peritonitis and Catheter-Related Infection Rates among Q1-2018, Q1-2019, and Q1-2020

**Figure 3:**
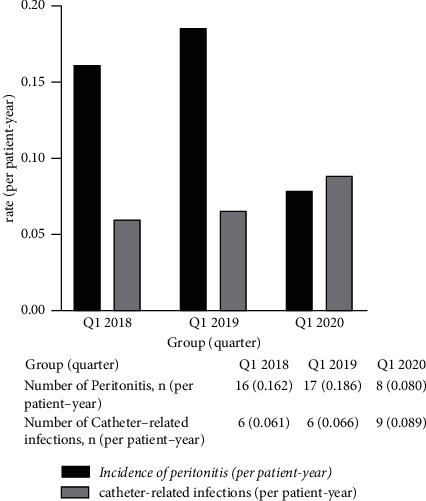
Comparison of peritoneal dialysis-related infections (Q1-2018: the first quarter of 2018; Q1-2019: the first quarter of 2019; Q1-2020: the first quarter of 2020).

The Q1-2020 quarter was associated with a significant decline in the incidence of peritonitis, compared with Q1-2018 and Q1-2019 (*χ*2 = 12.782, *P* < 0.001; *χ*2 = 18.960, *P* < 0.001, respectively), with no significant difference in catheter-related infection rates among the three groups (all *P* > 0.05, Figure 3).

#### 3.1.5. Comparison between Outpatient Follow-Up and Remote Follow-Up PD Patients of Q1-2020

There were no COVID-19 infections from patients treated at the Peritoneal Dialysis Center. No significant differences were observed for clinical data, such as gender, age, and body mass index between outpatient and remote follow-up patients (*P* > 0.05, Table 3). No significant differences were observed for any blood indices between the two groups (all *P* > 0.05, Table 4). No significant differences were observed between the two groups for any indexes (all *P* > 0.05, Table 5).

## 4. Discussion

It was reported that COVID-19 infection presents a particular threat to patients on dialysis [11, 12]. Preliminary studies confirmed that patients on maintenance hemodialysis are susceptible to COVID-19, as are patients on peritoneal dialysis [13]. To prevent the COVID-19 infection, our center issued new policies, including disseminating propaganda, enhancing education, and changing follow-up methods, consistent with strategies adopted in many countries [14, 15]. It is reported that a portion of COVID-19 critically ill patients who developed acute renal failure underwent emergency bedside PD, which had significant advantages compared to intermittent hemodialysis or variations of continuous renal replacement therapy [16, 17]. This study found that telehealth positively impacted patient outcomes during the COVID-19 pandemic.

Although outpatient follow-up continued to represent the predominant follow-up type, telehealth in the management of PD patients gets progressive attention. Remote follow-up by telephone or Internet-based platforms represented a significantly increased proportion of follow-up types during the COVID-19 pandemic. In place of conventional face-to-face contact between patients and doctors, telehealth follow-up can provide health education remotely, eliminating geographical isolation between patients and hospitals [18]. Diverse remote-monitoring platforms exist to record patients' vital signs, daily body weight, ultrafiltration (UF), and target values. Dialysis treatment data can significantly predict dialysis-related complications and prognosis [19], especially during automated peritoneal dialysis (APD) patients' telehealth management. APD usage can provide clinicians with the ability to identify and intervene in peritoneal dialysis-related problems at early stages, reducing the hospitalization rate and the incidence of clinical complications, improving patients' quality of life [20]. European and American countries use APD much more frequently than China, with preponderant APD remote-monitoring systems, whereas this treatment remains to be developed in China. In this study, during the COVID-19 pandemic, PD patients were suggested to use remote follow-up methods to avoid COVID-19 infection. Peritoneal dialysis staffers of the hospital followed up with patients and their families using telephone or Internet-based platforms to provide basic knowledge, and preventive measures regarding COVID-19 provide appropriate suggestions for health, nutrition, and spiritual support. This is the most important reason for the dramatic increase in remote follow-up patients. Although telehealth for home dialysis cannot wholly replace on-site treatment, it can effectively avoid possible infection risks and provide psychosocial support for patients during COVID-19, which is of great benefit to both doctors and patients [21].

This study found a decline in the hospitalization rate during the COVID-19 pandemic. Hospitalization control measures were launched during this period to prevent cross-patient infections. The decline also benefited from remote communications, which provided patient self-management support, including health information, patient education, telephone support, and support group participation. Standardized self-management support may represent an effective tool for slowing advanced chronic kidney disease progression, reducing hospitalization events, and maximizing clinical efficacy [22]. Besides, the reduction of peritonitis discussed in detail below also plays a role in declined hospitalization.

The present study also found that the drop-out rate during COVID-19 was lower than during the first quarter of 2019. The pandemic directly restricted patient movement, confining them to their homes, thereby indirectly reducing exposure to respiratory and gastrointestinal pathogens. Besides, patients strengthened their protective behaviors, such as washing hands and wearing masks, and significantly reduced the risks of exposure to infection. Consequently, peritonitis-associated drop-out events reduced, accompanied by a significant reduction in the incidence of peritonitis. Finally, remote patient management continued to facilitate the early detection and management PD-related problems, contributing to reduced hospitalization and clinical complications.

In this study, the incidence of peritonitis was significantly lower during Q1-2020 than during Q1-2018 and Q1-2019. Peritonitis is a common complication of peritoneal dialysis and represents the primary cause of peritoneal dialysis technical failure [23]. The Guidelines of the International Society for Peritoneal Dialysis (ISPD) primarily attribute peritonitis to issues involving handwashing and mask-wearing [24], and strengthening patient training may be a critical factor in the prevention of peritoneal dialysis-related infections. Patient training hinges on hand hygiene. All patients must receive aseptic technical training, learn how to handle contamination, and participate in regular retraining, encompassing how to perform dialysis exchange procedures, wash hands, identify the symptoms and signs of peritonitis, discern contamination and respond appropriately, and nurse after discharge. Besides, patients learn to avoid potential risk factors, such as hypoproteinemia, vitamin D deficiency, depression, incorrect connection methods, technical errors, hypokalemia, long-acting antibiotics, medical procedures, constipation, colonization and infections at the exit site, and contact with pets, to mitigate the risk of peritonitis [25]. During the COVID-19 pandemic, the hospital offered remote services to patients, conducted by peritoneal dialysis nurses, to enhance patient self-protection by guiding patients in correct handwashing and mask-wearing procedures, strictly observing aseptic operations, regularly verifying whether patients were following recommendations, and strengthening the retraining and self-management of patients. The risk of peritonitis was also effectively reduced by minimizing outdoor activities, avoiding unnecessary contact with public surfaces and public transportation, and reducing contact and gathering activities with social groups. Because the outbreak of COVID-19 coincided with Chinese New Year, a festival associated with an increase in eating outside of the home, COVID-19 patients may also suffer from gastrointestinal infections and be more likely to suffer enterogenous peritonitis due to overeating and an unhealthy diet. Thus, eating at home and reducing outdoor activities were advocated, reducing enterogenous peritonitis.

In this study, the rate of catheter-related infections appears to have increased in Q1-2020 compared to prior years. Usually, the exit/tunnel infection related to PD catheters was evaluated by medical staff. A lower or prolonged in-hospital visit may delay the PD catheter exit management and cause exit/tunnel infection in PD patients.

### 4.1. Limitations

According to the current clinical analysis, the COVID-19 period was not associated with significant differences in patients' blood index levels at the Peritoneal Dialysis Center compared with the same period during the previous two years. However, the decrease in the outpatient follow-up rate resulted in a decline in the clinical data collected; therefore, the possibility of unstated data and consequent errors must be acknowledged. Besides, because fewer patients were willing or able to attend given COVID-19 risks, catheter infection may be underestimated in this study. Another limitation is that patients themselves based-bias could not be omitted for evaluation of our results. Finally, the method of remote follow-up was not recorded, which is also a shortage of this study. Because of the inherent disadvantage of this retrospective single-center study, the results should be verified by multicenter analyses that recruit large samples of patients.

## 5. Conclusion

During the COVID-19 pandemic (Q1-2020), increased remote follow-up was practiced in our center's PD patients. The hospitalization rate and peritonitis incidence were significantly decreased compared with the same time in previous years. This study showed that telehealth methods might be a reasonable way to manage PD patients. Large-scale, multicenter studies must confirm these results.

## Figures and Tables

**Table 1:** Comparison of general data in Q1-2018, Q2-2019, and Q1-2020.

Group (quarter)	Number of cases	Gender (male/female)	Age (years)	BMI (kg/m^2^)	Dialysis vintage (months)
Q1-2018	394	221/173	56.3 ± 15.1	22.05 ± 3.31	43.8 ± 33.4
Q1-2019	366	194/172	57.4 ± 14.6	22.55 ± 3.09	50.3 ± 35.7
Q1-2020	401	213/188	57.9 ± 14.6	22.51 ± 3.47	52.0 ± 39.0
*P*	—	0.568	0.278	0.064	0.004

Q1-2018: the first quarter of 2018; Q1-2019: the first quarter of 2019; Q1-2020: the first quarter of 2020; BMI: body mass index (body mass (kg)/height (m)^2^).

**Table 2:** Comparison of serological indicators in Q1-2018, Q2-2019, and Q1-2020.

Indicators	Q1-2018	Q1-2019	Q1-2020	*P*
Hemoglobin (g/L)	113.4 ± 17.7	112.7 ± 18.1	115.9 ± 18.4	0.074
Calcium (mmol/L)	2.24 ± 0.21	2.26 ± 0.21	2.27 ± 0.19	0.195
Phosphorus (mmol/L)	1.74 ± 0.53	1.70 ± 0.48	1.70 ± 0.51	0.423
iPTH (pg/mL)	386.2 ± 299.3	344.4 ± 264.5	377.9 ± 361.2	0.207
Albumin (g/L)	37.6 ± 5.3	37.0 ± 4.8	37.8 ± 5.6	0.183
Urea (mmol/L)	18.8 ± 5.2	19.1 ± 6.5	19.1 ± 11.5	0.918
Creatinine (*μ*mol/L)	937.6 ± 312.9	941.9 ± 436.9	901.9 ± 290.5	0.322
Uric acid (*μ*mol/L)	430.7 ± 91.4	422.2 ± 244.8	406.2 ± 96.1	0.173
Potassium (mmol/L)	4.09 ± 0.72	4.07 ± 0.73	4.11 ± 0.74	0.809

Q1-2018: the first quarter of 2018; Q1-2019: the first quarter of 2019; Q1-2020: the first quarter of 2020; iPTH: intact parathyroid hormone. ANOVA has been used in the comparison of serological indicators.

**Table 3:** Comparison of general data between outpatient and remote follow-up patients.

General data	Outpatient follow-up (*n* = 287)	Remote follow-up (*n* = 114)	*P* value
Males/females (%)	150 (52.3%)/137 (47.7%)	63 (55.3%)/51 (44.7%)	0.588
Mean age, yr	57.2 ± 14.3	57.6 ± 14.4	0.812
BMI (kg/m^2^)	22.33 ± 3.38	22.66 ± 3.17	0.380
Dialysis vintage (months)	39.2 ± 33.1	39.3 ± 30.7	0.986

PD: peritoneal dialysis; Q1-2020: the first quarter of 2020; BMI: body mass index (body mass (kg)/height (m)^2^).

**Table 4:** Comparison of serological indicators between outpatient and remote follow-up patients.

Observation Indicators	Outpatient follow-up (*n* = 287)	Remote follow-up (*n* = 114)	*P*
Hemoglobin (g/L)	114.50 ± 19.26	115.88 ± 19.05	0.530
Calcium (mmol/L)	2.28 ± 0.22	2.28 ± 0.20	0.906
Phosphorus (mmol/L)	1.67 ± 0.57	1.78 ± 0.50	0.096
iPTH (pg/mL)	337.46 ± 256.55	397.17 ± 331.46	0.073
Albumin (g/L)	36.40 ± 5.72	37.52 ± 4.84	0.095
Urea (mmol/L)	19.44 ± 10.79	21.18 ± 14.97	0.203
Creatinine (*μ*mol/L)	892.07 ± 304.29	912.66 ± 301.25	0.547
Uric acid (*μ*mol/L)	405.60 ± 112.39	414.90 ± 91.99	0.446
Potassium (mmol/L)	4.07 ± 0.75	4.10 ± 0.65	0.675

iPTH: intact parathyroid hormone.

**Table 5:** Comparison of hospitalization rate, drop-out rate, and PD-associated infection.

Observation indicators	Outpatient follow-up patients (*n* = 287)	Remote follow-up patients (*n* = 114)	Pearson chi-squared test *χ*^2^	*P* value
Number of hospitalization, *n* (%)	45 (15.7%)	18 (15.8%)	0.001	0.978
Number of drop out, *n* (%)^*∗*^	6 (2.09%)	3 (2.63%)	0.109	0.718
Number of peritonitis, *n* (per patient-year)^*∗*^	4 (0.056)	4 (0.140)	1.867	0.231
Number of catheter-related infections, *n* (per patient-year)^*∗*^	5 (0.070)	4 (0.140)	1.161	0.281

^*∗*^Fisher' precision probability test.

## Data Availability

The data used in this study are available from the corresponding author upon request.

## References

[1] Huang C., Wang Y., Li X. (2020). Clinical features of patients infected with 2019 novel coronavirus in Wuhan, China. *The Lancet*.

[2] Chinese Center for Disease Control and Prevention Epidemiology Working Group for NCIP Epidemic Response (2020). The epidemiological characteristics of an outbreak of 2019 novel coronavirus diseases (COVID-19) in China. *Zhonghua Liu Xing Bing Xue Za Zhi = Zhonghua Liuxingbingxue Zazhi*.

[3] Shahzad F., Shahzad U., Fareed Z., Iqbal N., Hashmi S. H., Ahmad F. (2020). Asymmetric nexus between temperature and COVID-19 in the top ten affected provinces of China: a current application of quantile-on-quantile approach. *The Science of the Total Environment*.

[4] Jiang H.-J., Tang H., Xiong F. (2020). COVID-19 in peritoneal dialysis patients. *Clinical Journal of the American Society of Nephrology*.

[5] Ronco C., Manani S. M., Giuliani A., Tantillo I., Reis T., Brown E. A. (2020). Remote patient management of peritoneal dialysis during COVID-19 pandemic. *Peritoneal Dialysis International: Journal of the International Society for Peritoneal Dialysis*.

[6] Wilkie M., Davies S. (2020). Peritoneal dialysis in the time of COVID-19. *Peritoneal Dialysis International: Journal of the International Society for Peritoneal Dialysis*.

[7] Li P. K.-T., Szeto C. C., Piraino B. (2010). Peritoneal dialysis-related infections recommendations: 2010 update. *Peritoneal Dialysis International: Journal of the International Society for Peritoneal Dialysis*.

[8] Habach G., Bloembergen W. E., Mauger E. A., Wolfe R. A., Port F. K. (1995). Hospitalization among United States dialysis patients: hemodialysis versus peritoneal dialysis. *Journal of the American Society of Nephrology*.

[9] Tanaka M., Ishibashi Y., Hamasaki Y. (2020). Hospitalization for patients on combination therapy with peritoneal dialysis and hemodialysis compared with hemodialysis. *Kidney International Reports*.

[10] Li P. K.-T., Szeto C. C., Piraino B. (2016). ISPD peritonitis recommendations: 2016 update on prevention and treatment. *Peritoneal Dialysis International: Journal of the International Society for Peritoneal Dialysis*.

[11] Naicker S., Yang C.-W., Hwang S.-J., Liu B.-C., Chen J.-H., Jha V. (2020). The novel coronavirus 2019 epidemic and kidneys. *Kidney International*.

[12] Jung H. Y., Lim J. H., Kang S. H. (2020). Outcomes of COVID-19 among patients on in-center hemodialysis: an experience from the epicenter in South Korea. *Journal of Clinical Medicine*.

[13] Xiong F., Tang H., Liu L. (2020). Clinical characteristics of and medical interventions for COVID-19 in hemodialysis patients in wuhan, China. *Journal of the American Society of Nephrology*.

[14] Ikizler T. A., Kliger A. S. (2020). Minimizing the risk of COVID-19 among patients on dialysis. *Nature Reviews Nephrology*.

[15] Alberici F., Delbarba E., Manenti C. (2020). Management of patients on dialysis and with kidney transplantation during the SARS-CoV-2 (COVID-19) pandemic in brescia, Italy. *Kidney International Reports*.

[16] Vigiola Cruz M., Bellorin O., Srivatana V., Afaneh C. (2020). Safety and efficacy of bedside peritoneal dialysis catheter placement in the COVID-19 era: initial experience at a New York city hospital. *World Journal of Surgery*.

[17] Sourial M. Y., Sourial M. H., Dalsan R. (2020). Urgent peritoneal dialysis in patients with COVID-19 and acute kidney injury: a single-center experience in a time of crisis in the United States. *American Journal of Kidney Diseases*.

[18] Krishna V. N., Managadi K., Smith M., Wallace E. (2017). Telehealth in the delivery of home dialysis care: catching up with technology. *Advances in Chronic Kidney Disease*.

[19] Wallace E. L., Rosner M. H., Alscher M. D. (2017). Remote patient management for home dialysis patients. *Kidney International Reports*.

[20] Milan Manani S., Rosner M. H., Virzì G. M. (2019). Longitudinal experience with remote monitoring for automated peritoneal dialysis patients. *Nephron*.

[21] Lew S. Q., Wallace E. L., Srivatana V. (2021). Telehealth for home dialysis in COVID-19 and beyond: a perspective from the American society of Nephrology COVID-19 home dialysis subcommittee. *American Journal of Kidney Diseases*.

[22] Chen S.-H., Tsai Y.-F., Sun C.-Y., Wu I.-W., Lee C.-C., Wu M.-S. (2011). The impact of self-management support on the progression of chronic kidney disease—a prospective randomized controlled trial. *Nephrology Dialysis Transplantation*.

[23] Bender F. H., Bernardini J., Piraino B. (2006). Prevention of infectious complications in peritoneal dialysis: best demonstrated practices. *Kidney International*.

[24] Dong J., Chen Y. (2010). Impact of the bag exchange procedure on risk of peritonitis. *Peritoneal Dialysis International: Journal of the International Society for Peritoneal Dialysis*.

[25] Akoh J. A. (2012). Peritoneal dialysis associated infections: an update on diagnosis and management. *World Journal of Nephrology*.

